# Brainstem Anesthesia During Retrobulbar Block: An Eye-Opener Clinical Case

**DOI:** 10.7759/cureus.66294

**Published:** 2024-08-06

**Authors:** Teresa Sanchez, Joana Rodrigues

**Affiliations:** 1 Anesthesiology, Unidade Local de Saúde de São José, Lisbon, PRT

**Keywords:** ophtalmic regional anesthesia, abcde approach, subarachnoid injection, brainstem anesthesia, retrobulbar block

## Abstract

The use of a retrobulbar anesthetic block for surgery of the posterior chamber is a common, effective, and safe practice, although not without risks. This clinical case aims to describe one of the most feared complications of this ophthalmic block, which demands a high degree of suspicion and agility for proper diagnosis and management.

A 91-year-old female patient, physical status ASA III, presents for vitrectomy via pars plana of the left eye due to retinal detachment. Light sedoanalgesia was performed, as well as a left retrobulbar block with 5 mL of local anesthetic. Approximately two minutes after the injection of the local anesthetic, she developed a sudden clinical decline of consciousness, accompanied by bilateral photoplegic mydriasis, sinus tachycardia, and hypertension, followed by central apnea. Orotracheal intubation and connection to a ventilatory prosthesis were performed, maintaining adequate oxygenation, ventilation, and hemodynamic stability. No abnormal findings were found in complementary diagnostic methods. The condition progressively reversed, with a gradual return to the initial state of consciousness, and it was possible to successfully extubate the patient after four hours. The patient remained stable, under surveillance, and was discharged home after 48 hours with no neurological impairment or ophthalmological complications.

The clinical findings are compatible with brainstem anesthesia, explained by the dispersion of the local anesthetic into the subarachnoid space, through an inadvertent puncture of the ophthalmic artery or the meninges that involve the optic nerve. Although this event is a rare complication, a low threshold of suspicion should be maintained, given the potential severity of the clinical condition. Early recognition should be followed by a systematic A-B-C-D-E approach, and the outcomes are often favorable. Careful surveillance and monitoring should accompany the performance of ophthalmic surgical procedures, and the presence of an anesthesiologist is essential for the quality of the services provided and patient safety.

## Introduction

The current clinical case was previously presented as a Poster at the 2022 Sociedade Portuguesa de Anestesiologia Annual Scientific Meeting on June 4, 2022.

The increasing number of ophthalmological surgeries requiring local and regional anesthesia should be followed by human and material resources that enable patient monitoring and safety, aiming to reduce perioperative morbimortality, particularly in an increasingly elderly population with significant comorbidities.

The performance of local and regional anesthesia for ophthalmological procedures is a common, effective, and safe practice, providing painless surgeries and akinetic eyes, avoiding general anesthesia-related complications, and promoting faster recovery after surgery. However, it is not harmless, and associated risks are well-known.

Traditionally, the gold standard of eye nerve blocks was retrobulbar anesthesia, which consists of injecting small volumes of a local anesthetic (3-5 mL) in the intraconal retrobulbar space. Nevertheless, the invasiveness and complications related to this technique made it obsolete, and others, namely peribulbar anesthesia (extraconal retrobulbar block) and sub-tenon's block are nowadays considered standard-of-care [1-7].

Regardless of the technique used, the potential complications can be divided into two subgroups: minor complications (sub-conjunctival hemorrhage and chemosis) and major sights and life-threatening complications, such as orbital hemorrhage, globe perforation, nerve injury, muscle palsy, oculocardiac reflex, allergy to local anesthetic agents and hyaluronidase, and severe systemic adverse events. The incidence of severe systemic adverse events has been reported as 3.4/10,000, most of them due to the spread of local anesthetic to the optic nerve sheath, leading to subarachnoid block, seizures, and brainstem anesthesia [4].

While certain anatomical characteristics may heighten the likelihood of complications, the primary risk factor stems from the operator's limited experience, leading to needle misplacement. In addition, the needle size and length are also important contributors. The 25 Gauge size and a maximum length of 25 mm are currently preferred by the majority of professionals [4].

## Case presentation

A 91-year-old female patient, physical status American Society of Anesthesiologist (ASA) III, presents for vitrectomy via pars plana of the left eye due to retinal detachment. The patient was partially dependent on daily life activities and had a past medical history of well-controlled hypertension, cardiac insufficiency NYHA II (mild shortness of breath and limitation during ordinary activities), and obesity (BMI: 35 kg/m^2^), medicated with furosemide 20 mg/day. Preoperative complementary diagnostic exams, namely blood analysis, chest radiograph, and electrocardiogram revealed no alterations.

The procedure started with dilation of the left pupil with 1% atropine sulfate eyedrops, positioning in dorsal decubitus, peripheral vein puncture with an 18G catheter, and ASA-standard monitoring. Oxygen at a rate of 2.5 L/min was administered by nasal catheter. The initial parameters were peripheric oxygen saturation of 97%, blood pressure (BP) of 137/85 mmHg, and heart rate (HR) of 89 bpm in sinus rhythm. Light sedoanalgesia was performed, with 20 μg of fentanyl and 0.625 mg of droperidol. After sedation and disinfection, a left intraconal retrobulbar block was performed by an experienced ophthalmologist, with a 30 mm, 25G needle and injection of 2.5 mL of 1% ropivacaine and 2.5 mL of 2% lidocaine.

**Figure 1:**
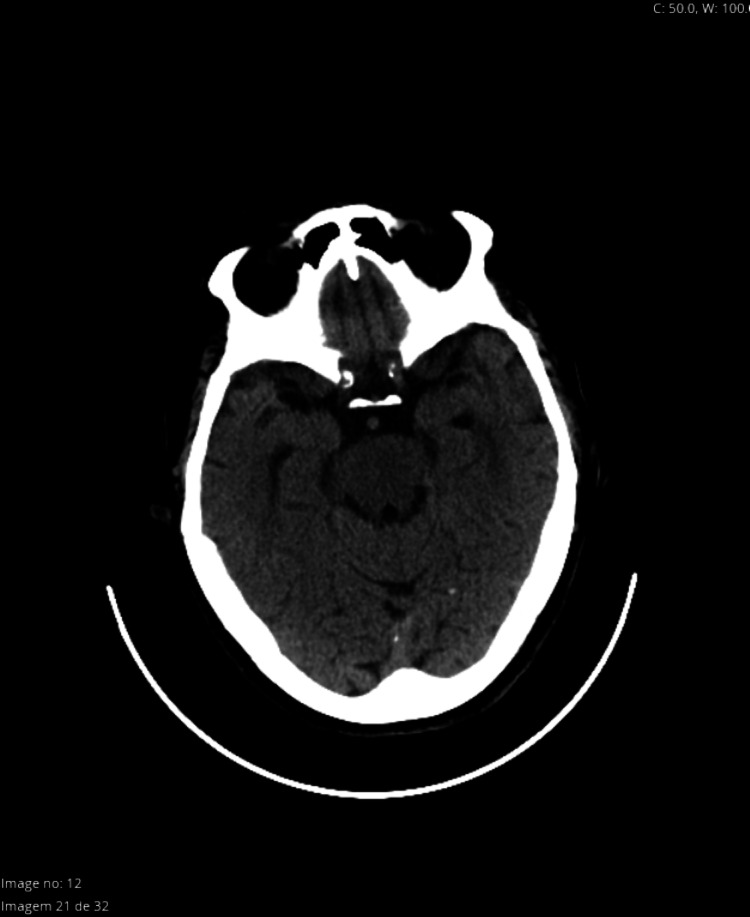
Cranioencephalic computed tomography appears normal

**Figure 2:**
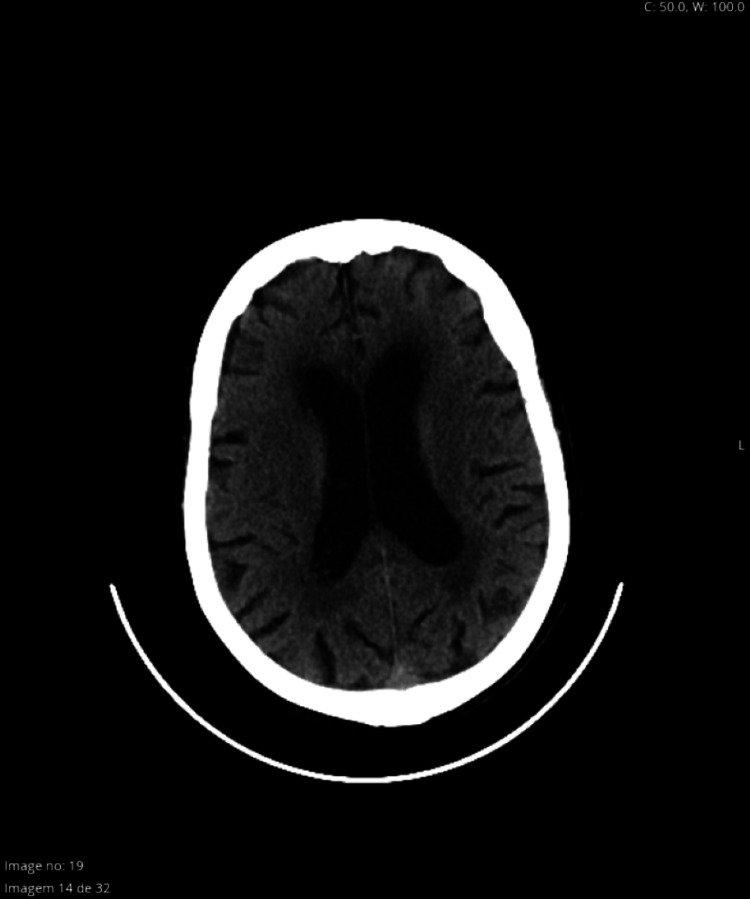
Cranioencephalic computed tomography shows no alterations

Approximately two minutes after the injection of the local anesthetic solution, the patient became agitated, followed by a rapid decline of consciousness with a Glasgow Coma Score 3, accompanied by bilateral photoplegic mydriasis, sinus tachycardia of 150 bpm and hypertension (BP: 225/100 mmHg), followed by central apnea. After manual ventilation with a face mask and 100% oxygen, it was monitored the anesthetic depth with Bispectral Index™ (BIS™), oscillating between 40 and 50. The electroencephalogram pattern was characterized by slow delta waves. It was administered propofol 20 mg, the patient was intubated under direct laryngoscopy and connected to the ventilatory prosthesis, maintaining adequate oxygenation and ventilation. The surgery was postponed, and her next of kin was informed of the occurrence. The patient remained hemodynamically stable and was first transferred to the emergency department for further evaluation and posteriorly to an intensive care unit (ICU) for surveillance. No sedation or cardiovascular support was required during the process. Complementary diagnostic methods were carried out, namely cranioencephalic computed tomography and blood analysis, which revealed no alterations (Figures 1-2). The condition progressively reversed, with a gradual return to the initial state of consciousness, spontaneous movement of four members, eye-opening and ventilatory stimulus and it was possible to successfully extubate the patient four hours later. The patient remained stable, under surveillance, and was discharged home after 48 hours, without any neurological deficits or ophthalmological complications.

## Discussion

Presented with a clinical picture of a sudden change in the state of consciousness with a decrease in the neurological score followed by respiratory arrest, a differential diagnosis was made, and hypotheses such as excessive sedation were put forward. After checking the minimum dose of opioid administered, this hypothesis was ruled out. According to the surgical team, the technique was performed uneventfully, without any difficulties or complications. The probability of a stroke following a hypertensive spike was also excluded, not only by the imaging tests carried out, namely a cranioencephalic CT scan but also by the complete and spontaneous remission of the condition without any permanent neurological deficits. Intra-arterial injection of local anesthetic could have been considered a possible mechanism since 15% of the population presents with the inferior ophthalmic artery located inferior to the optic nerve and is therefore more prone to inadvertent puncture. Intra-arterial injection of local anesthetic produces a retrograde flow to the internal carotid arteries, affecting the thalamus and other midbrain structures. Although associated with an altered state of consciousness, respiratory arrest and hemodynamic instability, the predominant sign is, however, the immediate onset of grand mal convulsive activity, which the patient did not present.

The clinical feature is compatible with brainstem anesthesia, explained by the dispersion of the local anesthetic into the subarachnoid space through inadvertent puncture of the meninges that involve the optic nerve, leading to brainstem and control center toxicity, compromising consciousness, ventilation, and hemodynamic stability. Usually, the first sign is a parasympathetic blockade and sympathetic hyperactivity, followed by confusion, coma, cranial nerve palsies, and central apnea, which the patient presented.

Despite anatomical variations, the inadequate performance of the technique is responsible for the majority of ophthalmic block complications. Furthermore, the choice of technique is paramount, considering that peribulbar and sub-tenon's blocks are effective and safer options compared to intraconal retrobulbar blocks. Lesion of the optic nerve, perforation of the eye, retrobulbar hemorrhage, and injection of local anesthetic in the sheath of the optic nerve have been described as potential complications, and more commonly, although not exclusively, with the retrobulbar block.

Although this is a rare complication, a low threshold of suspicion should be maintained, given the potential severity of the clinical condition. Early recognition should be followed by a systematic A-B-C-D-E approach, and the evolution, in most cases, is favorable, with complete remission of symptoms within a few hours, requiring in most cases no more than support treatment. Depending on the condition, if a respiratory arrest is present, it requires oxygen administration and controlled invasive ventilation.

There is no unanimity regarding the adequate timing to perform the proposed surgery. Since the clinical picture should be promptly diagnosed, it has been described as successful and non-complicated surgeries performed before the complete remission of symptoms. In our clinical case, despite the most likely hypothesis being brainstem anesthesia, and since the surgeons denied any difficulty or critical step outside the routine in performing the block, it was necessary to resort to further investigation, namely to rule out a stroke. Thus, the procedure was postponed. When contacted after discharge, the patient no longer intended to undergo surgery.

## Conclusions

The performance of ophthalmic anesthetic blocks for eye surgery is not risk-free, and all members of the operating theater team should be aware of the potential complications and have regular training in basic life support. Peripheric vein cannulation is mandatory with peribulbar and retrobulbar blocks and when intra-operative sedation is used. Favoring an extraconal approach, as well as finer and shorter needles, are considered safer options. Careful surveillance and standard ASA monitoring should accompany the performance of ophthalmic surgical procedures, and the presence of an anesthesiologist is paramount for the quality of services provided and patient safety.
